# Neoadjuvant chemotherapy followed by definitive local treatment in locally advanced sinonasal squamous cell carcinoma

**DOI:** 10.3389/fonc.2024.1488066

**Published:** 2024-10-28

**Authors:** Jina Kim, Min Hee Hong, Hye Ryun Kim, Sun Min Lim, Chang Gon Kim, Da Hee Kim, Nam Suk Sim, Hyun Jun Hong, Yoon Woo Koh, Se-Heon Kim, Chan Woo Wee, Chang Geol Lee, Ki Chang Keum, Chang-Hoon Kim, Kyung Hwan Kim

**Affiliations:** ^1^ Department of Radiation Oncology, Yonsei Cancer Center, Heavy Ion Therapy Research Institute, Yonsei University College of Medicine, Seoul, Republic of Korea; ^2^ Division of Medical Oncology, Department of Internal Medicine, Yonsei Cancer Center, Yonsei University College of Medicine, Seoul, Republic of Korea; ^3^ Department of Otorhinolaryngology, Severance Hospital, Yonsei University College of Medicine, Seoul, Republic of Korea

**Keywords:** sinonasal cancer, neoadjuvant chemotherapy, concurrent chemoradiotherapy, surgery, prognosis

## Abstract

**Background:**

Sinonasal squamous cell carcinoma (SCC) is a rare disease entity, comprising less than 5% of malignancies of the head and neck. While surgery is the primary treatment approach, neoadjuvant and adjuvant therapies play crucial roles in enhancing the prognosis of patients undergoing treatment with the goal of cure. In this study, we aimed to explore the treatment outcomes of neoadjuvant chemotherapy (NAC) in patients with locally advanced sinonasal SCC.

**Methods:**

Medical records of patients diagnosed of locally advanced (cT3-4b, N0-3) sinonasal SCC treated with a definitive aim between January 2005 and March 2023 were retrospectively reviewed. Patients were categorized into the following groups based on the initial treatment: NAC followed by surgery, NAC followed by concurrent chemoradiotherapy (CCRT), definitive CCRT, or upfront surgery. Initial treatment plan was decided by a multidisciplinary team. Primary endpoint was overall survival (OS) and objective response rate, and secondary endpoints were progression free survival (PFS), cumulative incidence of local and distant failures, and treatment-related toxicity. The treatment response was assessed according to the RECIST criteria.

**Results:**

Total 126 patients were included, and the median follow-up period was 25.6 months. The objective response rate to NAC was 48.2%. The subsequent resection rate was 70%, 42.9%, and 16.7% for patients with stage T3, T4a, and T4b disease, respectively. Two-year progression-free survival did not differ significantly between the NAC followed by surgery and upfront surgery groups (53.6% vs. 60.6%, *P* = 0.615) or between the NAC followed by CCRT and definitive CCRT groups (26.7% vs. 37.4%, *P* = 0.506).

**Conclusion:**

NAC may be a valuable treatment option for patients with locally advanced sinonasal SCC, as it provides an opportunity for curative surgery and exhibits non-inferior oncological outcomes compared with upfront definitive local treatments.

## Introduction

Sinonasal squamous cell carcinoma (SCC) is a rare disease, accounting for less than 5% of all head and neck malignancies (1, 2). The maxillary sinus is the most common primary tumor site, followed by the nasal cavity (3, 4). Most patients are diagnosed at a locally advanced stage with tumors extending to adjacent critical structures such as the orbit or skull base, making treatment challenging (5, 6). While surgery is the primary treatment approach, multimodal therapies, including definitive radiotherapy (RT), concurrent chemoradiotherapy (CCRT), surgery followed by adjuvant RT, or neoadjuvant chemotherapy (NAC), can improve the prognosis of patients with sinonasal SCC (7, 8).

The use of NAC in locally advanced head and neck cancer has been explored with the aim of preventing micrometastases and distant metastasis, enabling evaluation of the response to chemotherapy and allowing tumor size reduction in patients with initially unresectable or borderline resectable tumors (9, 10). However, NAC may extend the overall treatment time, increase the risk of hematological toxicities, and lead to poor responders losing the chance to receive definitive local treatment (11, 12). The efficacy of NAC has previously been demonstrated in many types of head and neck cancers (13, 14). One previous study from the MD Anderson Cancer Center have compared the outcomes of CCRT versus surgery after NAC in patients with sinonasal undifferentiated carcinoma and found that CCRT may result in improved disease-specific survival in patients who showed favorable response to NAC (15). Nevertheless, due to the rarity of the disease, the effect of NAC in sinonasal SCC has not been fully explored previously.

In this study, we investigated the effects of NAC in locally advanced sinonasal SCC. Specifically, we compared the oncological outcomes of patients with locally advanced sinonasal SCC who underwent NAC followed by local treatment to those of patients who underwent upfront definitive CCRT or surgery.

## Materials and methods

### Study population

The medical records of 538 patients with sinonasal cancer who received treatment between January 2005 and March 2023 at a single referral institution were reviewed. The inclusion criteria were as follows: a) age ≥20 years at diagnosis, b) histologically confirmed SCC, c) locally advanced stage (cT3–4b, cN0–3), and d) received definitive treatment. Patients with distant metastasis at diagnosis, who received non-curative treatment, or were lost to follow-up were excluded. Finally, a total of 126 patients were included in the analysis. This study was approved by the Institutional Review Board of Yonsei University College of Medicine, Seoul, Republic of Korea (approval no. 4-2023-1337), which waived the requirement for informed consent owing to the retrospective nature of the study.

### Treatment and response evaluation

All patients underwent staging evaluation using computed tomography (CT), magnetic resonance imaging (MRI), and positron emission tomography/computed tomography, if available. Initial treatment plan was decided by a multidisciplinary team comprising radiologists, otorhinolaryngologists, medical oncologists, and radiation oncologists on an individual basis considering patients’ medical fitness and tumor resectability. Medically fit patients with resectable tumor underwent upfront surgery, while those with borderline resectable or unresectable tumor underwent either definitive CCRT or NAC. Medical fitness was defined as a status able to tolerate surgery under general anesthesia, considering patients’ compliance, performance status and comorbidities altogether. Among the patients who underwent NAC, those who showed response to NAC and were downstaged to resectable tumor later received surgery while those with no response underwent CCRT. Medically unfit patients received definitive CCRT (**Supplementary Figure 1**). Since 2018, NAC has been included as a viable option in management of sinonasal SCC in our institution in order to decrease the extent of surgical resection required and reduce borderline resectable or unresectable tumors to enable resection. Resectability of tumor was decided mostly regarding T stage, and the extent of surgery and choice of an endoscopic or open approach were at the discretion of the surgeon. Patients were categorized according to their initial treatment plan as follows: NAC followed surgery, NAC followed by CCRT, definitive CCRT, and upfront surgery. The patients in the NAC group received various combinations of chemotherapy, most of which included 5-fluorouracil, cisplatin, or docetaxel. All the patients in the upfront surgery group received postoperative RT with or without concurrent chemotherapy. CCRT typically involved weekly administrations of 30–40 mg/m^2^ cisplatin.

RT involved three-dimensional conformal or intensity-modulated RT. All patients underwent planning CT, and thermoplastic masks were used for immobilization. For patients receiving definitive RT, the gross tumor volume was defined as the primary lesion and any metastatic lymph nodes, as observed on CT or MRI. For patients undergoing postoperative RT, the gross tumor volume was defined as the surgical bed and any residual lesion. Clinical target volume (CTV) 1 was delineated to include microscopic disease by creating a 3–5 mm margin around the gross tumor volume. CTV2 was delineated by adding a second margin 5–10 mm outside CTV1, including the involved adjacent substructures. The planning target volume (PTV) was determined as a 2–3 mm uniform expansion from the CTV. In cases of elective nodal irradiation, ipsilateral level IB and II lymph nodes were commonly included; bilateral lymph nodes were included if the primary tumor crossed the midline. For patients receiving definitive RT, the median RT dose was 69.96 Gy (59.4–75 Gy in 1.8–3 Gy per fraction) and 54 Gy (30.6–64 Gy in 1.6–2.5 Gy per fraction) for PTV1 and PTV2, respectively. For patients receiving postoperative RT, the median RT dose was 64.05 Gy (48.6–70.4 Gy in 1.8–3 Gy per fraction) and 54 Gy (30.6–60 Gy in 1.6–2.5 Gy per fraction) for PTV1 and PTV2, respectively.

Follow-up imaging and laboratory analyses were performed every 3 months for the first 2 years after the initial treatment and every 3–6 months thereafter. The treatment response was assessed according to the Response Evaluation Criteria in Solid Tumors, and the objective response rate was defined as the proportion of patients with a complete or partial response.

Treatment-related toxicity was evaluated according to the Common Terminology Criteria for Adverse Events version 5.0. Acute toxicity was defined as toxicity arising during treatment or within 3 months of treatment completion, whereas late toxicity was defined as toxicity arising more than 3 months after treatment was completed.

### Statistical analysis

The primary endpoints were overall survival (OS) and overall response rate. The secondary endpoints included the progression-free survival (PFS), the cumulative incidence of local failure, the cumulative incidence of distant failure, and treatment-related toxicity. OS was defined as the time to death from any cause from the date of treatment initiation, whereas PFS was defined as the time to progression or death, whichever came first, from the date of treatment initiation. The Kaplan–Meier method and the log-rank test were used for survival analyses. The cumulative incidence of local and distant failures was estimated using Gray’s test. *P* < 0.05 was considered statistically significant. SPSS Statistics for Windows software version 25.0 (IBM Corp., Armonk, NY, USA) and R software version 4.0.3 (R Foundation for Statistical Computing, Vienna, Austria) were used for statistical analyses.

## Results

### Patient characteristics and treatment regimens

Patient characteristics and treatment regimens are summarized in **Table 1**. The number of patients who received NAC followed by surgery, NAC followed by CCRT, definitive CCRT, and upfront surgery was 14 (11.1%), 15 (11.9%), 42 (33.3%), and 54 (42.9%), respectively. The most frequently used chemotherapy regimen for NAC was a combination of docetaxel and cisplatin, followed by a combination of 5-fluorouracil, cisplatin, and docetaxel. The median number of NAC cycles was three (range, 1–6), and nine (30%) patients needed a 20–25% dose reduction. For patients receiving CCRT, weekly cisplatin was the most frequently used chemotherapy regimen. Among the 14 patients who underwent NAC followed by surgery, 11 (78.6%) received postoperative CCRT, and three (21.4%) received postoperative RT alone. Among the 54 patients in the upfront surgery group, 21 (38.9%) received postoperative CCRT, and 33 (61.1%) received postoperative RT alone. Of the 32 patients who received postoperative CCRT following either upfront surgery or NAC and surgery, 28 (87.5%) received 30–40 mg/m^2^ cisplatin every week, and one (3.1%) received 80 mg/m^2^ cisplatin every 3 weeks. One patient who did not respond to cisplatin during NAC received cetuximab. The most common primary tumor site was the maxillary sinus (n = 90, 71.4%), and most patients had N0 disease (n = 101, 80.2%). A higher proportion of patients in the upfront surgery group than in the other two groups had stage T3 and N0 disease; there were no differences in age or sex among the groups.

**Table 1:** Patient and treatment characteristics.

Variables	Total (n = 126)	NAC (n = 29)*		Def CCRT (n = 42)	Upfront Op (n = 54)	p value
		NAC → Op (n = 14)	NAC → CCRT (n = 15)			
Age, median (range), years	60 (21-88)	61 (35-76)	60 (21-84)	62 (33-88)	59 (36-80)	0.440
Sex, n (%)						0.866
Male	95 (75.4)	10 (71.4)	11 (73.3)	33 (78.6)	40 (74.1)	
Female	31 (24.6)	4 (28.6)	4 (26.7)	9 (21.4)	14 (25.9)	
Tumor site, n (%)						0.535
Maxillary sinus	90 (71.4)	12 (85.7)	12 (80.0)	30 (71.4)	35 (64.8)	
Nasal cavity	17 (13.5)	1 (7.1)	0	6 (14.3)	10 (18.5)	
Ethmoid sinus	15 (11.9)	1 (7.1)	3 (20.0)	5 (11.9)	6 (11.1)	
Frontal sinus	3 (2.4)	0	0	1 (2.4)	2 (3.7)	
Sphenoid sinus	1 (0.8)	0	0	0	1 (1.9)	
Clinical T stage, n (%)						0.004
T3	51 (40.5)	7 (50.0)	3 (20.0)	9 (21.4)	32 (59.3)	
T4a	48 (38.1)	6 (42.9)	8 (53.3)	20 (47.6)	14 (25.9)	
T4b	27 (21.4)	1 (7.1)	4 (26.7)	13 (31.0)	8 (14.8)	
Clinical N stage, n (%)						0.036
N0	101 (80.2)	9 (64.3)	12 (80.0)	30 (71.4)	50 (92.6)	
N1	10 (7.9)	1 (7.1)	2 (13.3)	5 (11.9)	2 (3.7)	
N2	14 (11.1)	4 (28.6)	1 (6.7)	6 (14.3)	2 (3.7)	
N3	1 (0.8)	0	0	1 (2.4)	0	
Concurrent chemotherapy						0.001
None	53 (42.1)	3 (21.4)	8 (53.3)	8 (19.0)	33 (61.1)	
Weekly Cisplatin	57 (45.2)	9 (64.3)	4 (26.7)	25 (59.5)	19 (35.2)	
Three-weekly Cisplatin	8 (6.3)	0	2 (13.3)	5 (11.9)	1 (1.9)	
Cetuximab	1 (0.8)	1 (7.1)	0	0	0	
Others	7 (5.6)	1 (7.1)	1 (6.7)	4 (9.5)	1 (1.9)	
Neoadjuvant chemotherapy						
Docetaxel+Cisplatin+5-FU	10 (33.3)	5 (35.7)	5 (33.3)			
Cisplatin+5-FU	3 (10.0)	0	3 (20.0)			
Docetaxel+Cisplatin	11 (36.7)	7 (50.0)	4 (26.7)			
TS-1+Cisplatin	2 (6.7)	1 (7.1)	1 (6.7)			
Others	4 (13.3)	1 (7.1)	2 (13.3)			
Surgery, n (%)						0.915
Endoscopic resection	30 (44.1)	6 (42.9)			24 (44.4)	
Open surgery	38 (55.9)	8 (57.1)			30 (55.6)	
Resection margin, n (%)						0.195
R0	19 (27.9)	2 (14.3)			17 (31.5)	
R1	25 (36.8)	4 (28.6)			21 (38.9)	
R2	24 (35.3)	8 (57.1)			16 (29.6)	
RT modality, n (%)						0.202
3D CRT	16 (12.8)	0	1 (6.7)	6 (14.3)	9 (16.7)	
IMRT	109 (87.2)	14 (100.0)	14 (93.3)	36 (85.7)	45 (83.3)	
Elective nodal irradiation, n (%)						0.388
No	49 (39.2)	2 (14.3)	8 (53.3)	14 (33.3)	25 (46.3)	
Yes	76 (60.8)	12 (85.7)	7 (46.7)	28 (66.7)	29 (53.7)	

NAC, neoadjuvant chemotherapy; Op, operation; CCRT; concurrent chemoradiotherapy; Def CCRT, definitive concurrent chemoradiotherapy; 5-FU, 5-fluorouracil; RT, radiotherapy; 3D CRT, three-dimensional conformal radiotherapy; IMRT, intensity-modulated radiotherapy.

*One patient who initially received NAC failed to receive further local treatment due to treatment related toxicity.

**Supplementary Figure 2** shows the initial treatment according to the T stage. The majority of patients with stage T3 disease underwent upfront surgery; the proportion of patients receiving NAC or definitive CCRT increased in those with stage T4 disease. One patient with stage T4b disease who received NAC failed to receive further definitive local treatment because of chemotherapy-induced grade 3 hematological toxicity.

### Treatment outcomes

**Figure 1:**
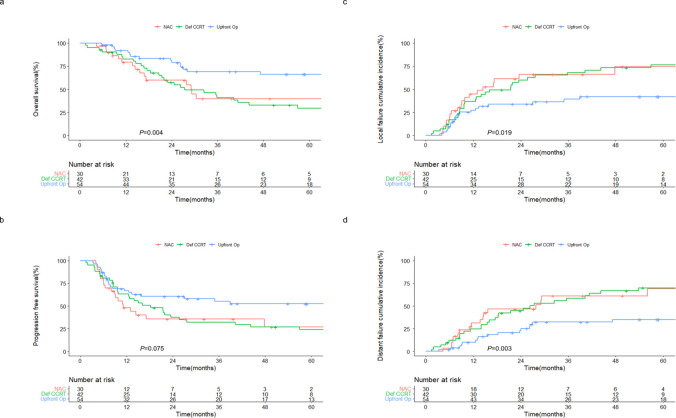
Outcomes of patients with sinonasal squamous cell carcinoma receiving different treatment regimens. Kaplan–Meier estimates of **(A)** overall and **(B)** progression-free survival, and cumulative incidences of **(C)** local and **(D)** distant failure in patients receiving NAC, definitive CCRT, and upfront surgery. NAC, neoadjuvant chemotherapy; Def CCRT, definitive concurrent chemoradiotherapy; op, operation.

Patients were followed up for a median period of 25.6 months. The 2-year OS rates of patients in the NAC, definitive CCRT, and upfront surgery groups were 59.7%, 57.4%, and 81.3%, respectively (*P* = 0.004) (**Figure 1A**). Similarly, the 2-year PFS rates were 35.6%, 37.4%, and 60.6% in the NAC, definitive CCRT, and upfront surgery groups, respectively (*P* = 0.075) (**Figure 1B**). The 2-year local failure rates were 66.2%, 60.1%, and 33.9% (*P* = 0.019; **Figure 1C**) and the 2-year distant failure rates were 46.7%, 44.7%, and 22.8% (*P* = 0.003; **Figure 1D**) in the NAC, definitive CCRT, and upfront surgery groups, respectively. No significant differences were observed in the OS, PFS, local failure, or distant failure rates between the NAC and definitive CCRT groups.

Subgroup analysis revealed 2-year OS rates of 60.0% and 64.3% (*P* = 0.607; **Supplementary Figure 3A**) and 2-year PFS rates of 26.7% and 53.6% (*P* = 0.325; **Supplementary Figure 3B**) in the NAC followed by CCRT and NAC followed by surgery groups, respectively. In addition, 2-year local failure rates were 73.3% and 51.0% (*P* = 0.298; **Supplementary Figure 3C**), and 2-year distant failure rates were 46.7% and 39.4% (*P* = 0.866; **Supplementary Figure 3D**), in the NAC followed by CCRT and NAC followed by surgery groups, respectively.

### NAC followed by definitive local treatment vs. upfront local treatment

**Figure 2:**
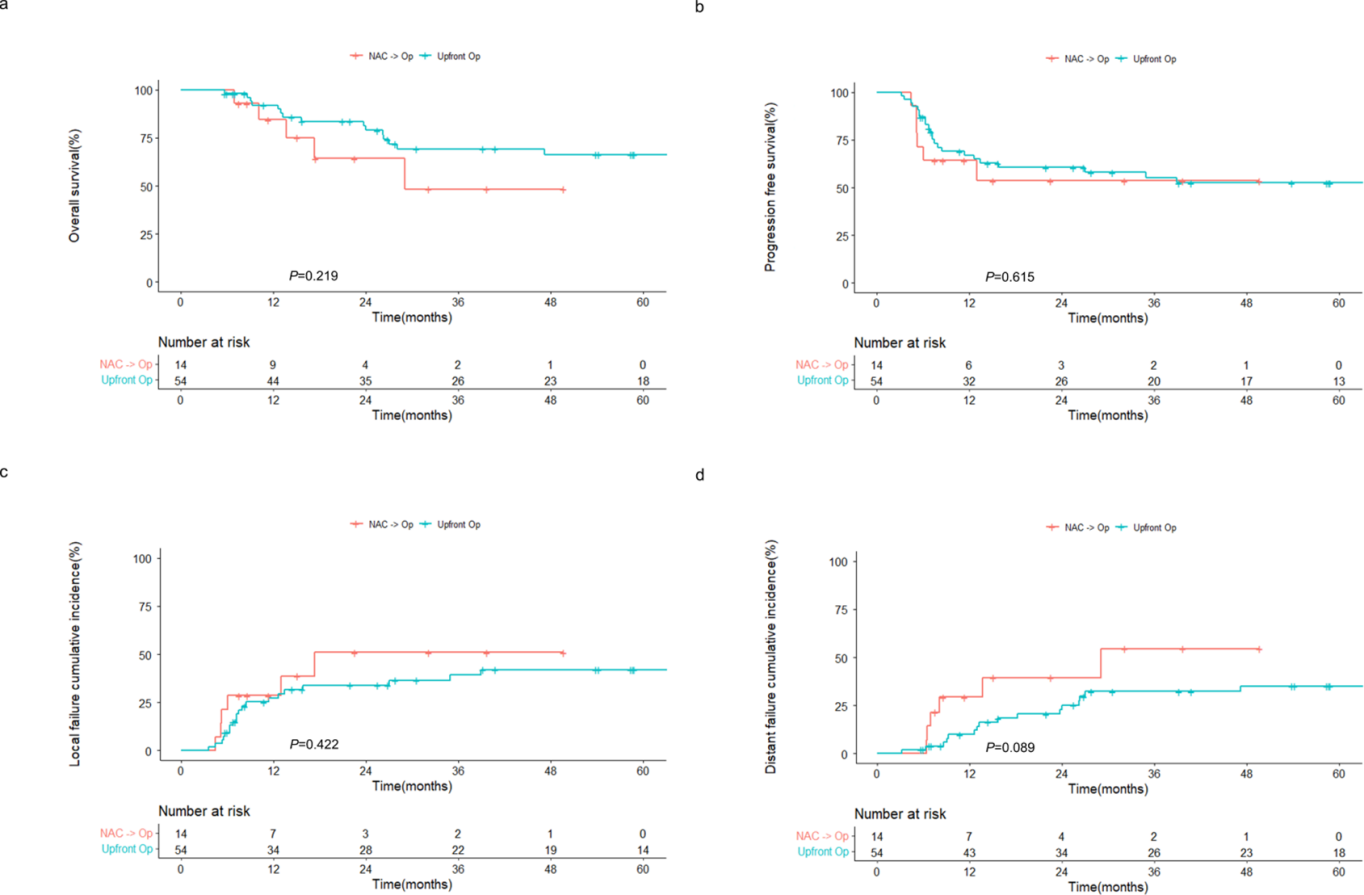
Outcomes of patients with sinonasal squamous cell carcinoma receiving surgery with or without prior NAC. Kaplan–Meier estimates of **(A)** overall and **(B)** progression-free survival, and cumulative incidences of **(C)** local and **(D)** distant failure in patients receiving NAC followed by surgery and upfront surgery. NAC, neoadjuvant chemotherapy; op, operation.

Subgroup analysis revealed that the OS and PFS rates of the NAC followed by surgery group were comparable to those of the upfront surgery group, despite patients undergoing NAC being typically at a more advanced stage and having higher R1-2 resection rates (85.7% vs. 68.5%). The 2-year OS rates were 64.3% and 81.3% (*P* = 0.219; **Figure 2A**) and the 2-year PFS rates were 53.6% and 60.6% (*P* = 0.615; **Figure 2B**) in the NAC followed by surgery and upfront surgery groups, respectively. Moreover, no significant differences in local (**Figure 2C**) and distant (**Figure 2D**) failures were observed between the two groups.

**Figure 3:**
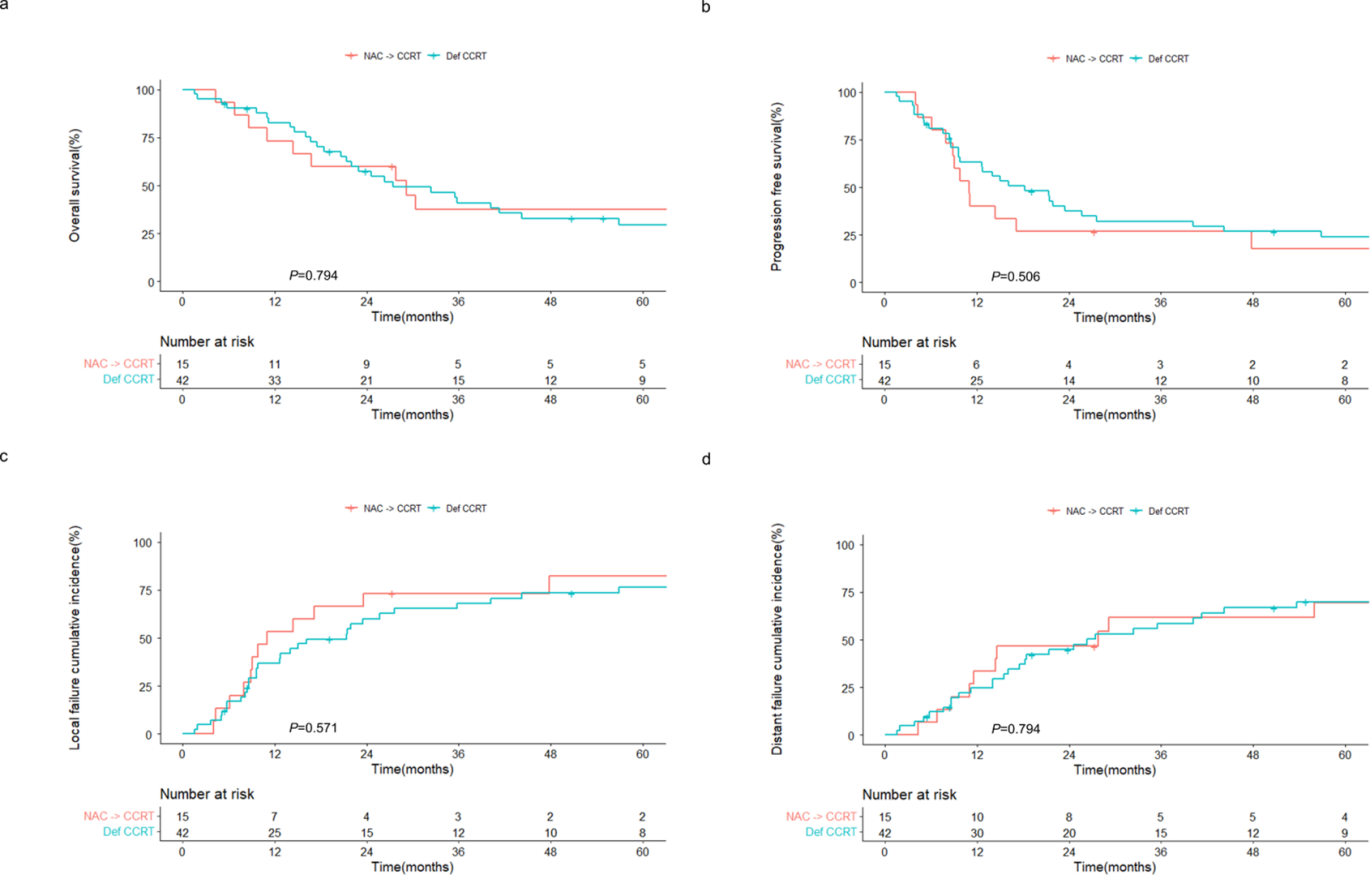
Outcomes of patients with sinonasal squamous cell carcinoma receiving CCRT with or without prior NAC. Kaplan–Meier estimates of **(A)** overall and **(B)** progression-free survival, and cumulative incidences of **(C)** local and **(D)** distant failure in patients receiving NAC followed by CCRT and definitive CCRT. NAC, neoadjuvant chemotherapy; CCRT, concurrent chemoradiotherapy; Def CCRT, definitive concurrent chemoradiotherapy.

Similarly, the outcomes of the NAC followed by CCRT group were comparable to those of the definitive CCRT group. The 2-year OS rates were 60.0% and 57.4% (*P* = 0.794; **Figure 3A**), and the 2-year PFS rates were 26.7% and 37.4% (*P* = 0.506; **Figure 3B**) in the NAC followed by CCRT and definitive CCRT groups, respectively. The cumulative incidences of local (**Figure 3C**) and distant (**Figure 3D**) failures also did not differ between the two groups.

### NAC treatment response and resection rate after NAC

Response rates to NAC are shown in **Supplementary Table 1**. Overall, an objective response was observed in 14 (48.2%) patients, including one (3.4%) who exhibited a complete response. Patients who showed a response to NAC had slightly improved OS and significantly improved PFS compared to those who did not show a response (2-year OS rates, 77.1% vs. 43.8%, *P* = 0.072, **Supplementary Figure 4A**; 2-year PFS rates, 56.3% vs. 16.4%, *P* = 0.031, **Supplementary Figure 4B**). The subsequent surgery rates were 57.1% and 50.0% in those who did and did not respond to NAC, respectively (*P* = 0.730). The rate of subsequent surgery decreased from 70.0% in patients at stage T3 to 16.7% in patients at stage T4b (*P* = 0.127; **Supplementary Table 2**). Among the 14 patients who underwent surgery following NAC, six (42.9%) were confirmed to have T-stage downstaging by pathology, and one (7.1%) had a pathological complete response (**Supplementary Table 3**).

### Treatment-related toxicity

The treatment-related toxicities are shown in **Table 2**. Acute grade 3 or 4 toxicity was more frequently reported in the NAC followed by surgery and NAC followed by CCRT groups than in the other groups (NAC followed by surgery, 21.4%; NAC followed by CCRT, 20.0%; definitive CCRT, 9.5%; upfront surgery, 5.6%; *P* = 0.145). Neutropenia was the most frequently reported form of acute toxicity in the NAC followed by surgery group (n = 9, 64.3%), while neutropenia and mucositis were the most frequently reported acute toxicities in the NAC followed by CCRT group (n = 5, 33.3% each). In the definitive CCRT group, the most commonly reported acute toxicity was mucositis (n = 22, 52.4%), followed by dermatitis (n = 7, 16.7%) and xerostomia (n = 7, 16.7%). Osteoradionecrosis was reported in two patients, one who received NAC followed by CCRT and one who received upfront surgery followed by adjuvant CCRT.

**Table 2:** Treatment related toxicities.

	NAC (n = 29)						Def CCRT (n = 42)			Upfront Op (n = 54)		
	NAC → Op (n = 14)			NAC → CCRT (n = 15)								
	Grade 1-2	Grade 3-4	Total	Grade 1-2	Grade 3-4	Total	Grade 1-2	Grade 3-4	Total	Grade 1-2	Grade 3-4	Total
**Any acute adverse event**	11 (78.6%)	3 (21.4%)	14 (100.0%)	8 (53.3%)	3 (20.0%)	11 (73.3%)	32 (76.2%)	4 (9.5%)	36 (85.7%)	43 (79.6%)	3 (5.6%)	46 (85.2%)
Dermatitis	1 (7.1%)			1 (6.7%)			7 (16.7%)		7 (16.7%)	13 (24.1%)		13 (24.1%)
Mucositis	7 (50.0%)			4 (26.7%)	1 (6.7%)	5 (33.3%)	21 (50.0%)	1 (2.4%)	22 (52.4%)	27 (50.0%)	2 (3.7%)	29 (53.7%)
Xerostomia	2 (14.3%)			2 (13.3%)			7 (16.7%)		7 (16.7%)	6 (11.1%)		6 (11.1%)
Anorexia	1 (7.1%)						3 (7.1%)		3 (7.1%)	2 (3.7%)		2 (3.7%)
Nausea	3 (21.4%)				1 (6.7%)		6 (14.3%)		6 (14.3%)	5 (9.3%)		5 (9.3%)
Vomiting	1 (7.1%)						1 (2.4%)		1 (2.4%)			
Dysgeusia	2 (14.3%)						2 (4.8%)		2 (4.8%)			
Hyposmia										1 (1.9%)		1 (1.9%)
Diarrhea				1 (6.7%)			1 (2.4%)		1 (2.4%)	1 (1.9%)		1 (1.9%)
Neuropathy	1 (7.1%)			1 (6.7%)						1 (1.9%)		1 (1.9%)
Skin rash	1 (7.1%)						1 (2.4%)		1 (2.4%)			
Conjunctivitis										2 (3.7%)		2 (3.7%)
Leukocytosis				1 (6.7%)								
Neutropenia	5 (35.7%)	4 (28.6%)	9 (64.3%)	3 (20.0%)	2 (13.3%)	5 (33.3%)	2 (4.8%)	2 (4.8%)	4 (9.5%)	1 (1.9%)		1 (1.9%)
Anemia	5 (35.7%)			7 (46.7%)			3 (7.1%)		3 (7.1%)	6 (11.1%)		6 (11.1%)
Thrombocytopenia	2 (14.3%)			1 (6.7%)			2 (4.8%)		2 (4.8%)	4 (7.4%)		4 (7.4%)
Otitis media		1 (7.1%)						1 (2.4%)	1 (2.4%)	3 (5.6%)	1 (1.9%)	4 (7.4%)
Otitis externa										1 (1.9%)		1 (1.9%)
**Any late adverse event**	2 (14.3%)	1 (7.1%)	3 (21.4%)	1 (6.7%)	1 (6.7%)	2 (13.3%)	9 (21.4%)	1 (2.4%)	10 (23.8%)	8 (14.8%)	1 (1.9%)	9 (16.7%)
Xerostomia							6 (14.3%)		6 (14.3%)	5 (9.3%)		5 (9.3%)
Skin rash							1 (2.4%)		1 (2.4%)			
Keratitis							1 (2.4%)		1 (2.4%)			
Osteoradionecrosis				1 (6.7%)						1 (1.9%)		1 (1.9%)
Tinnitus							1 (2.4%)		1 (2.4%)			
Otitis media		1 (7.1%)										
Cutaneous fistula					1 (6.7%)			1 (2.4%)	1 (2.4%)			
Dysgeusia	1 (7.1%)									1 (1.9%)		1 (1.9%)
Neuropathy	1 (7.1%)											
Nasolacrimal duct obstruction						1 (2.4%)		1 (2.4%)		1 (1.9%)	1 (1.9%)
Hyposmia							1 (2.4%)		1 (2.4%)	2 (3.7%)		

NAC, neoadjuvant chemotherapy; Op, operation; CCRT, concurrent chemoradiotherapy; Def CCRT, definitive concurrent chemoradiotherapy.

## Discussion

In this study, we investigated the efficacy of NAC in patients with locally advanced sinonasal SCC. NAC is used with the expectations that a favorable response can reduce tumor volume, increase resectability, and eradicate micrometastases. However, NAC can be disadvantageous in patients who respond only minimally to this therapy, for whom the chances of local control could be decreased by delaying definitive local treatment. In the present study, 14 of the 30 patients who received NAC underwent surgery, and 15 patients whose tumors remained unresectable underwent CCRT. Only one patient could not receive subsequent definitive local treatment. The treatment outcomes revealed that NAC did not compromise local control, even in patients who could not undergo surgery after NAC. Moreover, patients who underwent NAC followed by surgery exhibited similar treatment outcomes to those who underwent upfront surgery in terms of overall survival and distant metastasis free survival, despite the more advanced tumor stage and the lower rate of concurrent chemotherapy administration during adjuvant radiotherapy in the former group. Collectively, our data imply that NAC may be a favorable treatment option for patients with sinonasal SCC, offering an opportunity for surgical resection without compromising local tumor control compared with immediate local treatment.

Neoadjuvant treatment has been shown to prolong patient survival by decreasing disease burden and controlling micrometastases in several solid tumor types, including esophageal, rectal, and lung cancers (16–19). However, NAC delays definitive local treatment, such as surgery or RT, and may preclude this treatment in 5–21% of patients if the response to NAC is poor (16–20). In addition, an extended overall treatment time increases the risk of accelerated repopulation in head and neck cancer (21). Despite these limitations, our study showed that the treatment outcomes of the NAC followed by surgery group did not differ significantly from those of the upfront surgery group, and the treatment outcomes of the NAC followed by CCRT group were comparable to those of the definitive CCRT group. In other words, although the initiation of local treatment may have been relatively delayed in the NAC group, this did not result in poor local control, and only one patient could not receive subsequent local treatment. Although distant failure did not decrease in the NAC group, this is consistent with the findings of the DeCIDE trial (22). Therefore, further studies are necessary to develop more efficient treatment modalities that can decrease distant metastasis.

In the present study, surgical resection was achieved in 14 of the 30 patients who received NAC. Notably, resectability after NAC was associated with the initial T stage. While subsequent surgery was performed in 70% of patients with stage T3 disease, this rate decreased to 42.8% and 16.7% in patients with stage T4a and T4b disease, respectively. These findings are consistent with those of Nyirjesy et al., who reported that of 10 patients with T4a or T4b sinonasal SCC who received NAC, only three (30%) underwent subsequent surgery (23). Since far-advanced cases, such as those at the T4b stage, are unlikely to become resectable after NAC, the benefits of NAC should be weighed in these cases. Definitive CCRT has shown outcomes comparable to those of upfront surgery in patients with locally advanced sinonasal SCC (24). Thus, it may be better to proceed with upfront CCRT in patients with far-advanced disease.

The overall response rate to NAC was 48.2% in this study, and patients who responded to NAC had prolonged survival outcomes. Several other studies have also reported the response rates of patients with sinonasal cancers to NAC. Recently, Abdelmeguid et al. reported the outcomes of induction chemotherapy followed by definitive local therapy in patients with locally advanced sinonasal SCC. Among the 123 patients included in the study, 71 (57.8%) responded to induction chemotherapy, with six (4.9%) exhibiting a complete response (25). In the SINTART 1 study, which reported the outcomes of multimodal treatment in patients with operable sinonasal tumors, the overall response rate to induction chemotherapy was 54% across all histologic types (26). The SINTART 2 study, which involved unresectable sinonasal tumors, reported an overall response rate of 40% (27). Consistent with the findings of this study, many studies have reported that the response to NAC is related to overall prognosis. A retrospective study of 95 patients with sinonasal undifferentiated carcinoma showed that those who responded to NAC had significantly prolonged 5-year disease-specific survival probabilities compared with those who showed less than a partial response (15). A Japanese group investigated the prognostic impact of the response to NAC in patients with sinonasal SCC who underwent curative surgery. They observed that OS and disease-free survival were significantly improved in the effective NAC group compared with the less effective NAC group (28). The identification of a biomarker that can predict the response to NAC is therefore necessary, and several trials are currently investigating whether genetic or radiomic factors can predict the response to NAC (29).

The major limitation of this study is its retrospective nature. The treatment selection was not randomized, and the inherent selection bias within each treatment group means that caution is required when interpreting the data. However, given the rarity of sinonasal SCC, prospective randomized trials may be impractical. Our data, encompassing a relatively large number of patients, provides additional evidence regarding the efficacy and safety of NAC in this patient group, complementing the results of previous studies (15, 25–28). The follow-up period of the study was relatively short; the long-term outcomes of NAC require further evaluation in future studies.

In conclusion, our study demonstrates that NAC can lead to acceptable outcomes for patients with locally advanced sinonasal SCC. Compared with upfront definitive local treatments, the delay in local treatment does not appear to compromise tumor control, suggesting that NAC can be a viable treatment strategy. Although larger cohort studies with longer follow-up periods are necessary, the results of this study suggest that NAC is a feasible alternative treatment option for patients with initially unresectable or borderline resectable sinonasal SCC.

## Data Availability

The raw data supporting the conclusions of this article will be made available by the authors, without undue reservation.
