# Supplementary material for: Neoadjuvant chemotherapy followed by definitive local treatment in locally advanced sinonasal squamous cell carcinoma
Source: Front Oncol. 2024 Oct 28;14:1488066. doi: 10.3389/fonc.2024.1488066 (PMC11551057; doi:10.3389/fonc.2024.1488066)

| **Supplementary Table 1. Response to neoadjuvant chemotherapy** | | | | |
| --- | --- | --- | --- | --- |
|  | **T3** | **T4a** | **T4b** | **Total** |
| CR | 1 | 0 | 0 | 1 (3.4%) |
| PR | 4 | 7 | 2 | 13 (44.8%) |
| SD | 2 | 2 | 0 | 4 (13.8%) |
| PD | 3 | 5 | 3 | 11 (37.9%) |

Abbreviations: CR, complete response; PR, partial response;
SD, stable disease; PD, progressive disease

| **Supplementary Table 2. Proportion of patients who underwent subsequent surgery following neoadjuvant chemotherapy by initial T stage** | | | |
| --- | --- | --- | --- |
| **Initial T stage** | **Subsequent surgery** | **No subsequent surgery** | ***P* value** |
| T3 | 7 | 3 | 0.127 |
| T4a | 6 | 8 |  |
| T4b | 1 | 4 |  |

**Supplementary Table 3. Clinical and pathological stage in patients who underwent neoadjuvant chemotherapy followed by surgery.**

| Patient No. | Clinical stage | Pathological stage |
| --- | --- | --- |
| 1 | T3N0 | T3N0 |
| 2 | T4aN0 | T2N0 |
| 3 | T3N0 | T1N0 |
| 4 | T4aN1 | T4aN1 |
| 5 | T3N0 | T1N0 |
| 6 | T4aN0 | T4aN0 |
| 7 | T3N0 | T3N0 |
| 8 | T3N0 | T0N0 |
| 9 | T3N0 | T3N1 |
| 10 | T4bN0 | T4aN0 |
| 11 | T4aN2 | T4aN0 |
| 12 | T3N2 | T3N0 |
| 13 | T4aN2 | T4aN1 |
| 14 | T4aN2 | T2N0 |

**Supplementary Figure 1. Flow of initial treatment decision.**

Def CCRT, definitive concurrent chemoradiotherapy; NAC, neoadjuvant chemotherapy


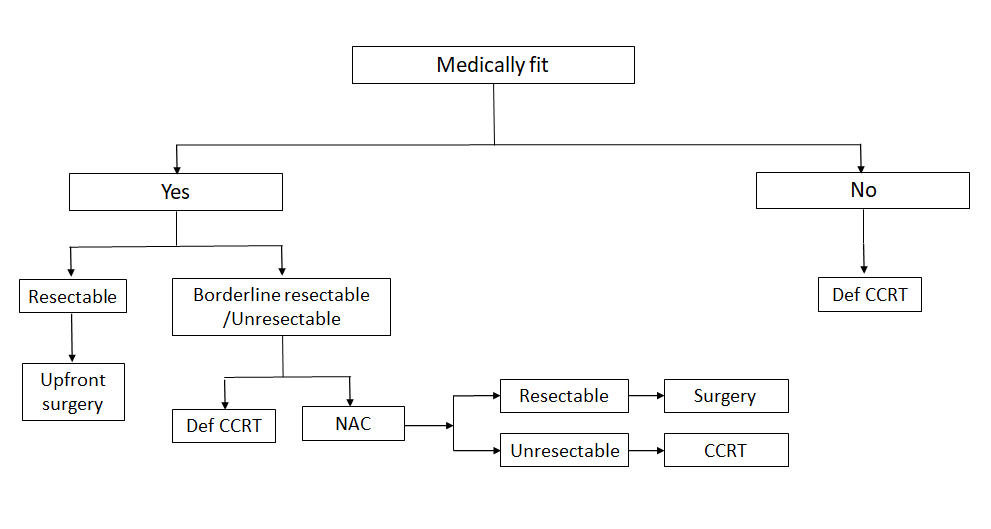


**Supplementary Figure 2. Treatment according to T stage.**

SCC, squamous cell carcinoma; Def CCRT, definitive concurrent chemoradiotherapy; CCRT, concurrent chemoradiotherapy; NAC, neoadjuvant chemotherapy; Op, operation; Tx, treatment

**
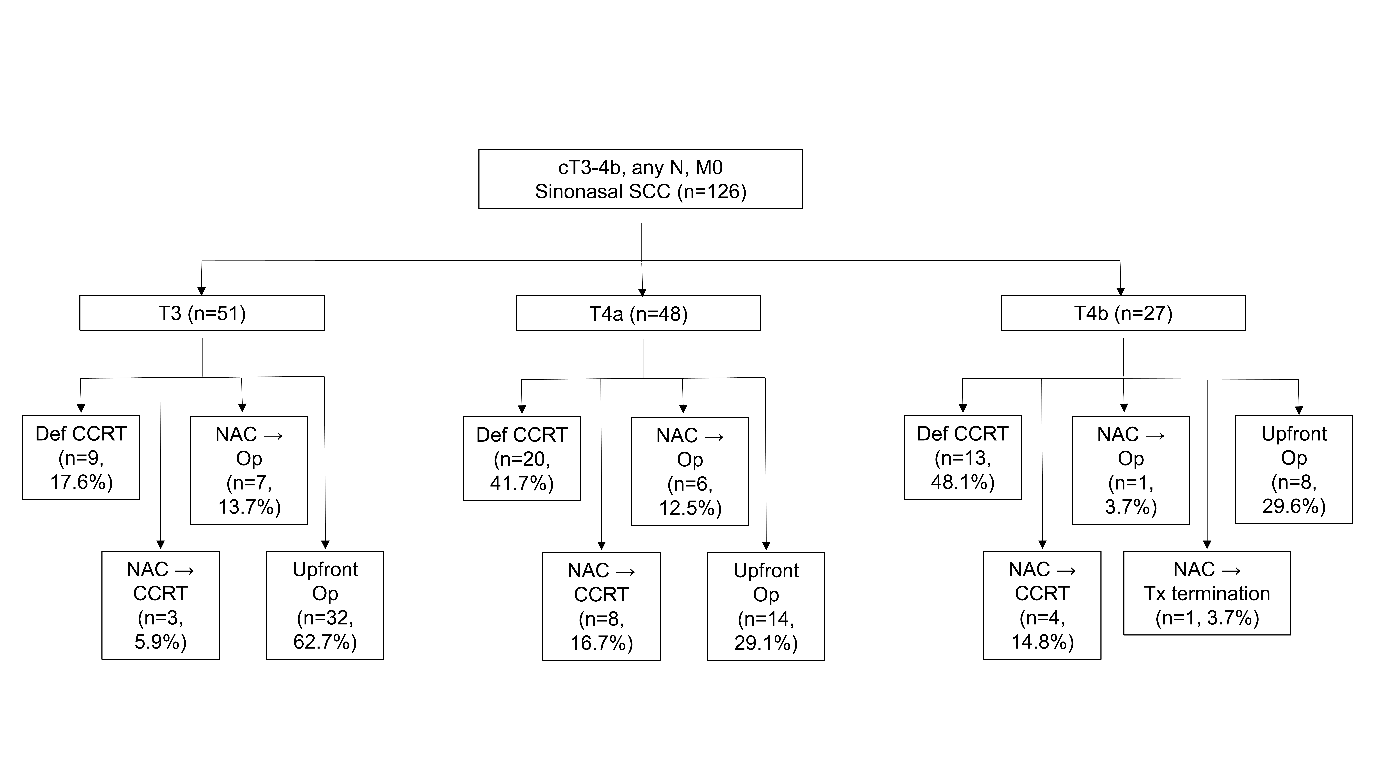
**

**Supplementary Figure 3. Outcomes of patients with sinonasal squamous cell carcinoma receiving NAC.**

Kaplan–Meier estimates of (A) overall and (B) progression-free survival, and cumulative incidences of (C) local and (D) distant failure in patients receiving NAC followed by CCRT and NAC followed by surgery.

NAC, neoadjuvant chemotherapy; CCRT, concurrent chemoradiotherapy; op, operation

**
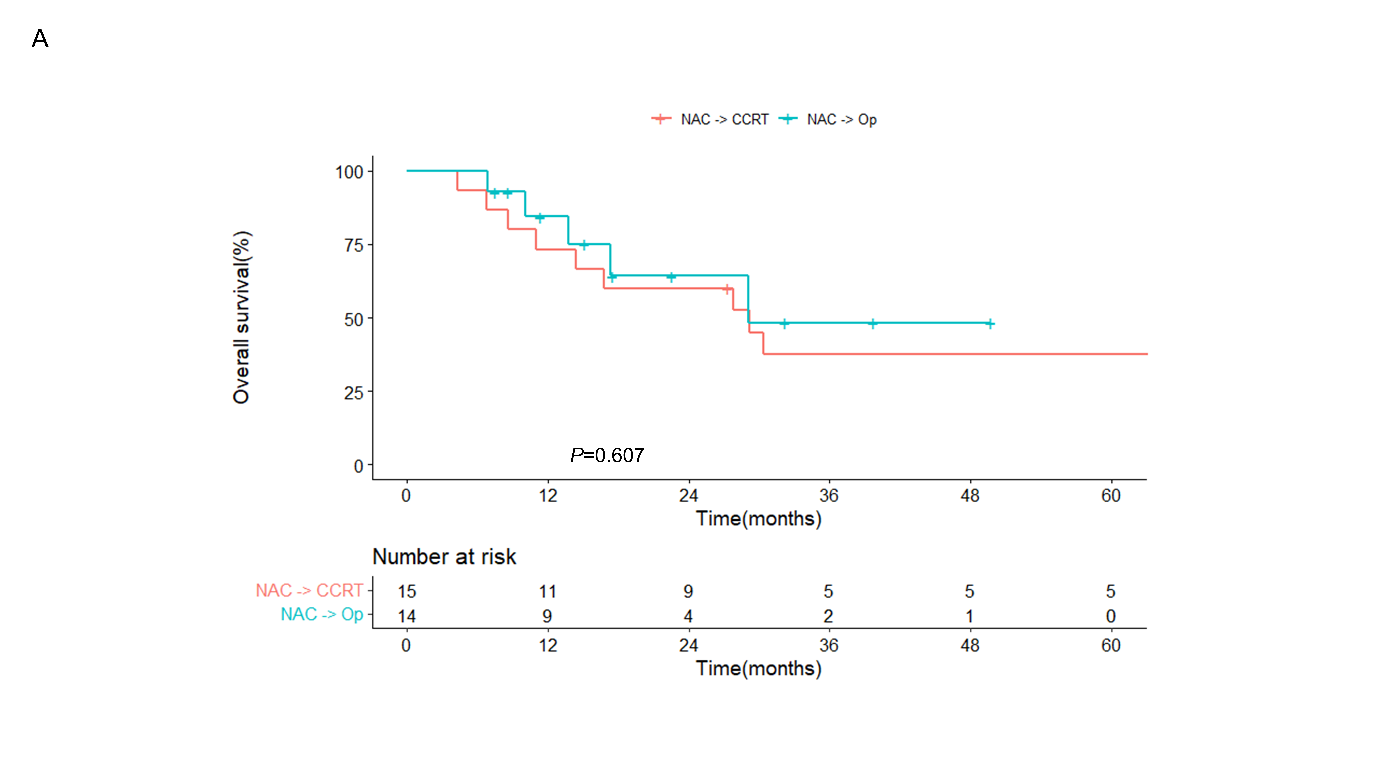
**

**
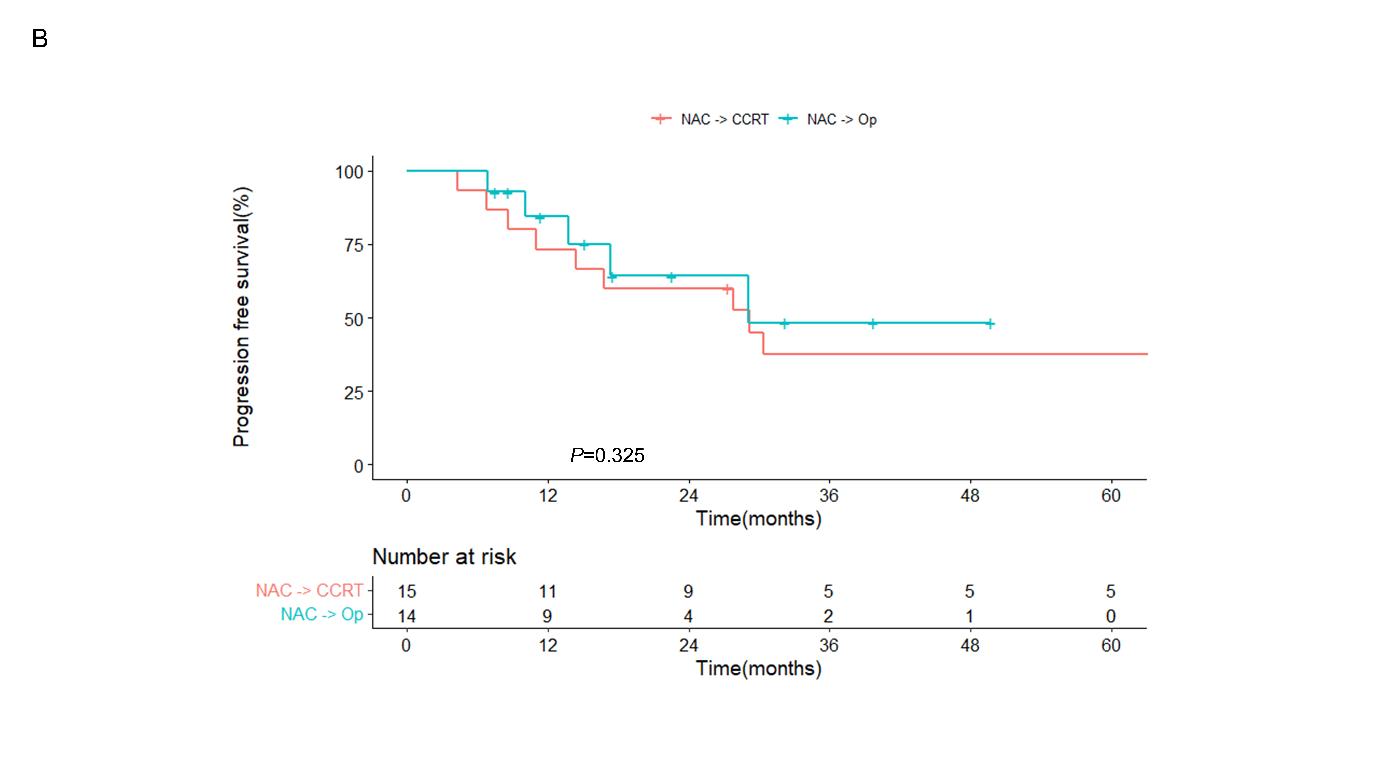
**

**
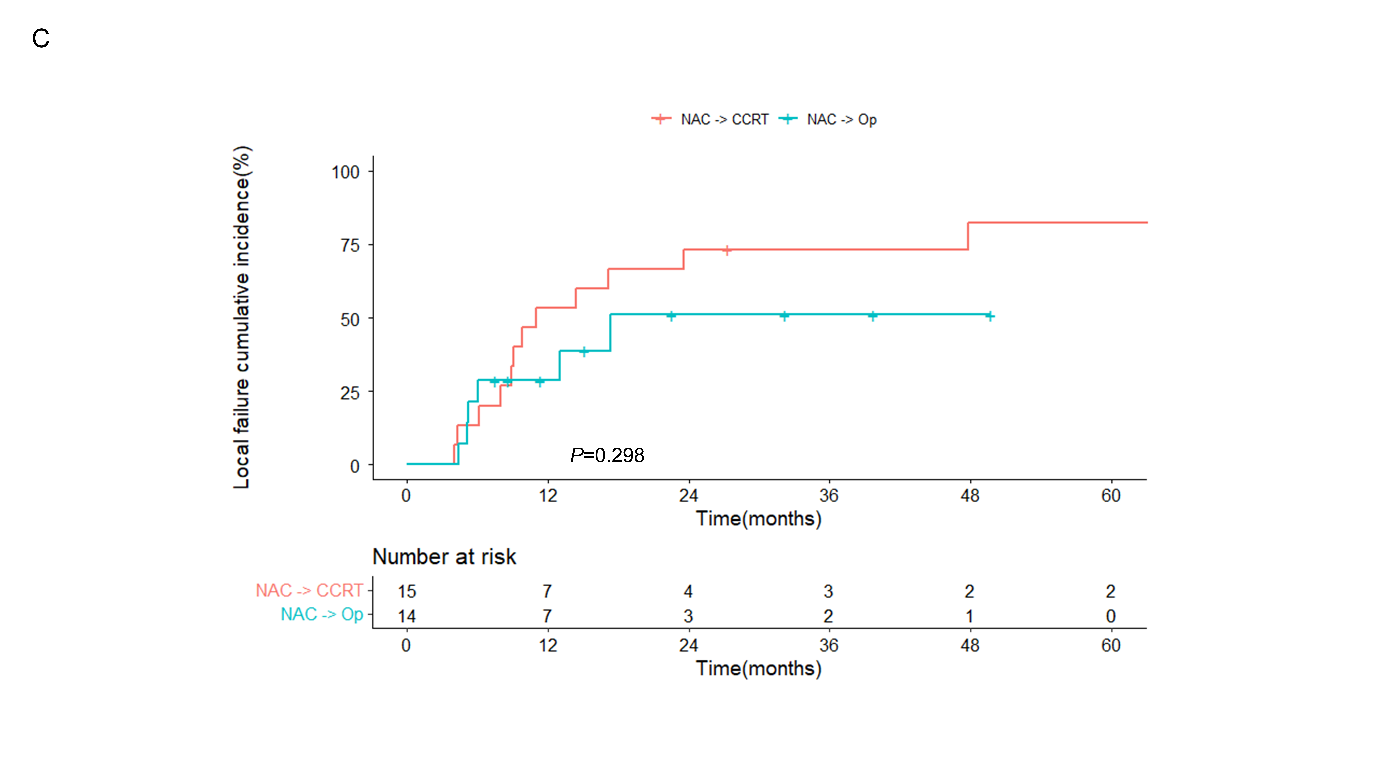
**

**
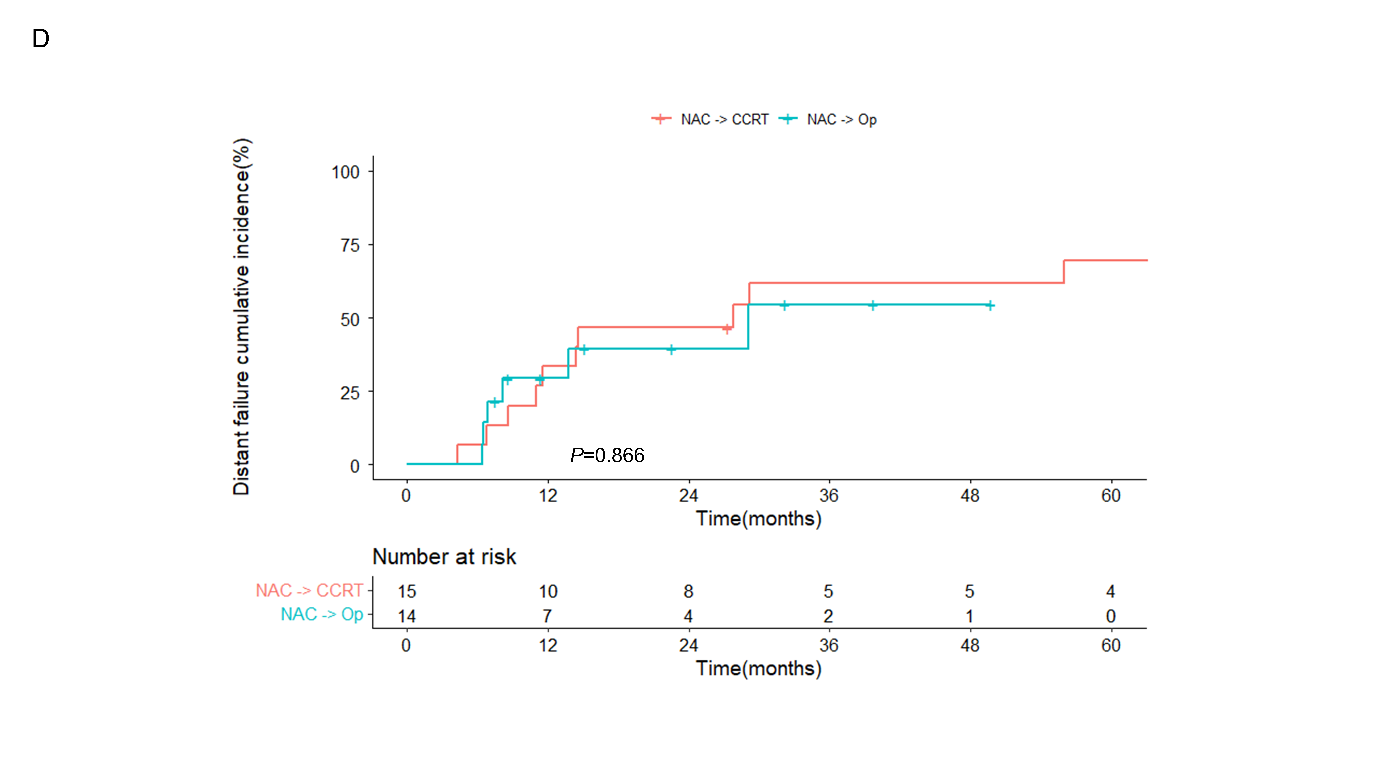
**

**Supplementary Figure 4.** **Outcomes of NAC responders and non-responders.**

Kaplan–Meier estimates of (A) overall and (B) progression-free survival of patients who did and did not respond to NAC.

NAC, neoadjuvant chemotherapy; CR, complete response; PR, partial response; SD, stable disease; PD, progressive disease.


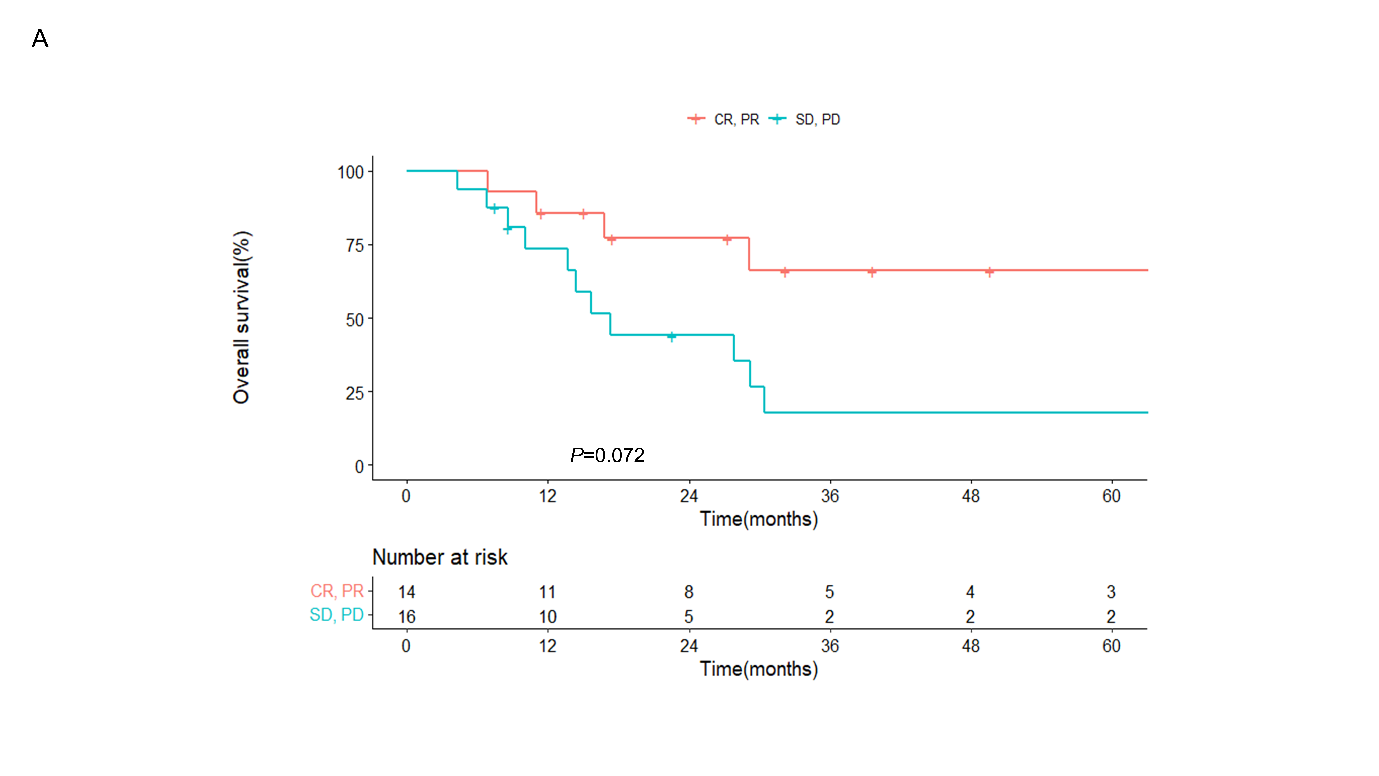


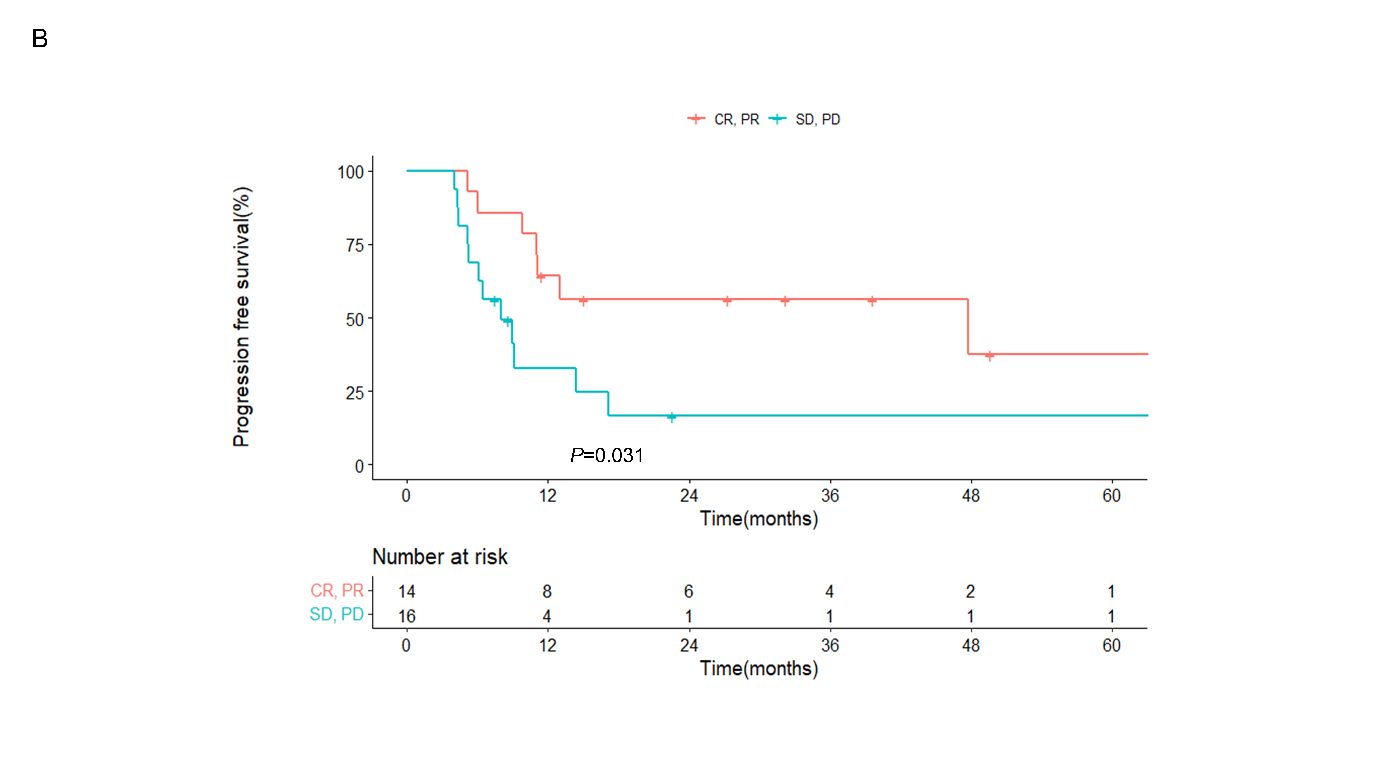

Supplement: Supplementary file 1 [file DataSheet1.docx]
